# Software-Defined Radio Implementation of a LoRa Transceiver

**DOI:** 10.3390/s24154825

**Published:** 2024-07-25

**Authors:** João Pedro de Omena Simas, Daniel Gaetano Riviello, Roberto Garello

**Affiliations:** Department of Electronics and Telecommunications (DET), Politecnico di Torino, 10129 Torino, Italy; joao.simas@usp.br (J.P.d.O.S.); daniel.riviello@polito.it (D.G.R.)

**Keywords:** Internet of Things, LoRa, software-defined radio, GNU Radio

## Abstract

The number of applications of low-power wide-area networks (LPWANs) has been growing quite considerably in the past few years and so has the number of protocol stacks. Despite this fact, there is still no fully open LPWAN protocol stack available to the public, which limits the flexibility and ease of integration of the existing ones. The closest to being fully open is LoRa; however, only its medium access control (MAC) layer, known as LoRaWAN, is open and its physical and logical link control layers, also known as LoRa PHY, are still only partially understood. In this paper, the essential missing aspects of LoRa PHY are not only reverse engineered, but also, a new design of the transceiver and its sub-components are proposed and implemented in a modular and flexible way using GNU Radio. Finally, some examples of applications of both the transceiver and its components, which are made to be run in a simple setup by using cheap and widely available off-the-shelf hardware, are given to show how the library can be used and extended.

## 1. Introduction

The main characteristics of low-power wide-area networks (LPWANs) are large coverage areas, low-power consumption (involving battery operated devices most of the time), and low data rates. The number of applications of this kind of technology has been growing in the past few years, especially with the rise of interest in Internet of Things (IoT) applications, in particular, related to the implementation of wireless sensor networks.

Despite this big interest in this field and the growing number of applications of LPWAN, a completely open protocol stack is still missing. The closest thing to it that is available at the moment is the LoRa protocol stack; however, only its upper layer, i.e., the MAC layer, is open. The LoRa Protocol stack, usually referred simply as LoRa, is usually subdivided into two parts: LoRa PHY, which, contrary to what the name implies, encompasses not only the physical layer aspects of this protocol stack, but also the logical link control (LLC) sublayer of the data link layer, and LoRaWAN, which comprises the medium access control sublayer of the Open Systems Interconnection (OSI) data link layer.

While LoRaWAN is open and publicly available [1], LoRa PHY is not. Some attempts have been made to reverse engineer it and propose designs for its demodulator [2,3,4,5,6,7], but some details are still not fully clear, which has prevented the implementation of a fully working, free, and open-source LoRa PHY transceiver.

In this paper, we continue previous work in the literature on reverse engineering the missing details of LoRa’s lower layers, and we propose an implementation of a fully free and open-source software-defined transceiver with the goal of allowing for further research and development to be carried out on this protocol stack by the scientific community. Also, this implementation is made to be modular to give more flexibility and ease the implementation of custom solutions at the lower levels to users of this technology. Finally, four example applications of the developed receiver and transmitter are given, both in terms of hardware and extra software. The first application illustrates how individual blocks can be used to implement simpler tasks, which do not require the full transmitter/receiver chain; it is a detector that can detect transmitted frames and infer which spreading factor (SF) and channel were used for the transmission and derive statistics in order to analyze the traffic in the network. The last three applications illustrate how the developed receiver and transmitter blocks can be used to implement more complex systems and extend GNU Radio functionalities.

The paper is organized as follows. In Section 2, we introduce LoRa. In Section 3, we present the reverse engineering of the LoRa physical layer, while in Section 4, we introduce a LoRa receiver structure. In Section 5, we address the GNU Radio transceiver implementation and the hardware setup, while in Section 6, we show some examples of applications. Finally, Section 7 contains our conclusions.

## 2. LPWAN and the LoRa Protocol Stack

In order to illustrate the context within which the LoRa protocol stack is situated in the framework of LPWAN, a brief description on some key topics is given in this section.

### 2.1. Low-Power Wide-Area Networks

Given that there is no standard definition for what exactly classifies as a LPWAN, it is not simply a network that covers a wide area and employs low-power devices. One attempt to define it, given the current applications that identify themselves as LPWAN, is the RFC8375 [8], published by the Internet Engineering Task Force (IETF). The following sums up their main characteristics:

Most technologies in this space aim for a similar goal of supporting large numbers of very low-cost, low-throughput devices with very low power consumption, so that even battery-powered devices can be deployed for years. LPWAN devices also tend to be constrained in their use of bandwidth (BW), for example, with limited frequencies being allowed to be used within limited duty cycles (usually expressed as a percentage of time per hour that the device is allowed to transmit). As the name implies, coverage of large areas is also a common goal. So, by and large, the different technologies are aimed at deployment in very similar circumstances.

Also, some important structural similarities among all the existing LPWAN “technologies”, a name given to protocol stacks combined with network topologies, are pointed out. Mainly, they are organized in terms of the following:

- End devices that communicate with Radio Gateways via a wireless link;
- Radio Gateway that connects to end devices using the LPWAN protocol and to a network gateway using Transmission Control Protocol (TCP) and Internet Protocol (IP);
- Network Gateway that connects the radio gateway to the internet (i.e., to an application server);
- Authentication Server that handles authentication, the joining of new devices to the network, and the assignment of encryption keys. This might be implemented in the same hardware as the Network Gateway.

Some of the most widely used LPWAN technologies are LoRa (i.e., LoRaWAN + LoRa PHY), Sigfox, and Narrowband IoT (NB-IoT). Table 1 shows a brief comparison of these three technologies in terms of their uplink communication characteristics in the physical, LLC, and MAC layers:

As it can be seen, although each of the three can be classified as LPWAN technologies, they have very distinct design paradigms. Sigfox takes a minimalistic approach, employing one of the simplest possible modulations, and making the hardware as equally simple, but ending up sacrificing bit rate.

NB-IoT is designed around the constraint of implementing an IoT LPWAN-oriented protocol stack, while reusing all the existing LTE standards as much as possible. This allows for existing components, software, and, more importantly, infrastructure built for LTE to be reused for NB-IoT. This constraint, however, also makes sure that NB-IoT inherits much of the complexity of the LTE protocol stack, which is arguably unnecessary in this context.

Finally, LoRaWAN is built with the main goal of being a flexible LPWAN protocol stack that takes advantage of the benefits of the LoRa CSS modulation. Since LoRaWAN is specifically designed for LPWAN applications, it results in having all the flexibility that Sigfox lacks without the complexity that comes with NB-IoT. Furthermore, it carries all the benefits that the CSS modulation has over the conventional ones.

The choice of working on LoRa for this paper was driven mainly by its flexibility and the fact that among the three IoT protocols, it is the only one whose lower layers (LoRaPHY) are neither public nor well known.

### 2.2. LoRaWAN

According to LoRaWAN’s specification [1], LoRaWAN is a MAC layer protocol made to run on top of the LoRa PHY physical layer, with the main goal of running networks of battery-powered end devices, which should run continuously for a long time.

In terms of network topology, usually a LoRaWAN network follows a “star-of-stars” topology with the usual organization described in the previous section on LPWAN (Section 2.1), where multiple gateways connect to a network server and the end devices connect to one or more gateways.

It is also important to note that LoRaWAN is designed to operate reliably on ISM bands which are to be shared with other devices using a variety of other protocols. To cope with this issue, in addition to the LoRa CSS modulation, it uses some MAC layer techniques, such as transmitting the message multiple times, hopping between channels in a pseudo-random fashion, and changing spreading factors, and consequently bit rates, in order to improve transmission robustness if needed.

Finally an important aspect of LoRaWAN is that it is uplink-focused, i.e., it gives special importance to messages between the end devices and the gateways; most of the MAC behavior is triggered by these. The main reflection of this fact is that in the most common form of operation (communication between A-class devices and gateways), downlink communication is only allowed in time slots located at fixed delays after the transmission of an uplink message.

### 2.3. LoRa Chirp Spread Spectrum Modulation

The CSS modulation used by LoRa PHY consists in using linear chirps as symbols; that is, signals with the form

(1) $$x_{i}\left(t\right)=A\exp\left\{j\pi \beta \;\mathrm{mod}{\left(t-T_{s}\frac{i}{N_{sym}},T_{s}\right)}^{2}+\varphi \right\},\;i\in \left\{0,\ldots ,N_{sym}\right\},t\in \left[0,T_{s}\right),$$

as shown in [15], where

- $T_{s}$ is the symbol period;
- β is a parameter which determines the speed of growth of the instantaneous frequency of that signal, which will be referred in the rest of the paper as the *chirp rate;*
- mod(a,b) is the real number remainder function defined as
  (2) $$\mathrm{mod}\left(a,b\right)=\min_{r\in R^{+}}\left\{a=qb+r,\;q\in Z\right\};$$
- $N_{sym}$ is the total number of symbols in the CSS modulation.

The parameters used by the LoRa PHY modulation are as follows:

- The mean bandwidth of the signal ($BW_{CSS}$), which relates with $T_{s}$ and β as
  (3) $$BW_{CSS}=\beta T_{s}.$$
  This can be set to a set of predefined values, described in  [16]; however, the LoRaWAN specification only uses 125 kHz and optionally 250 kHz for uplink messages and 500 kHz for downlink messages.
- The spreading factor is not only the number of bits that each symbol encodes, i.e, $N_{sym}=2^{SF}$, but it is also related to the previously mentioned parameters, such that
  (4) $$2^{SF}=BW_{CSS}T_{s}.$$

These signal sections have instantaneous frequency

(5) $$f_{i}\left(n\right)=\beta \;\mathrm{mod}\left(t-T_{s}\frac{i}{N_{sym}},T_{s}\right)\;$$

and the phase shift φ at each symbol is used to ensure the phase continuity of the signal.

**Figure 1:**
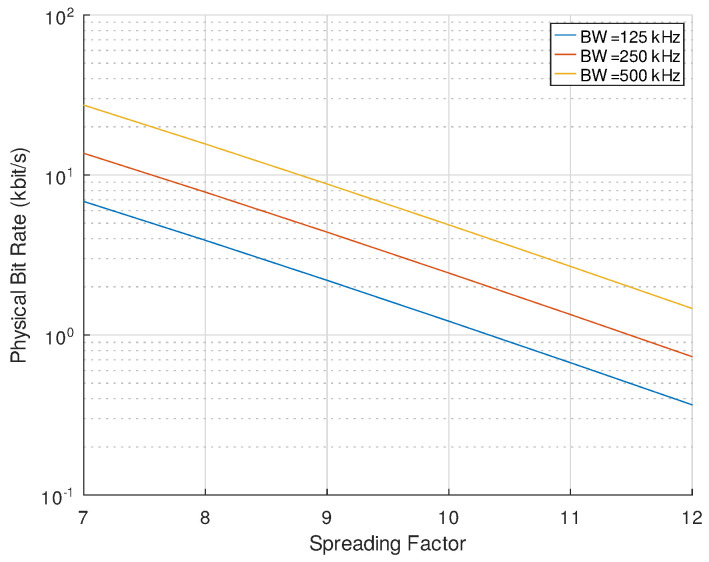
Physical bit rate in function of SF and BW for some of the possible values.

To illustrate the effects of these parameters, Table 2 and Figure 1 show the resulting physical bit rates with some of the possible parameter combinations, mainly the ones used in LoRaWAN:

## 3. Reverse Engineering the LoRa Physical Layer

### 3.1. Experimental Setup

To perform the following analysis, a combination of the data provided in [17] and data captured with a simple experimental setup was used.

**Figure 2:**
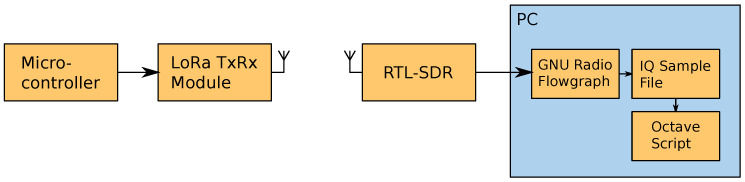
Diagram of the used experimental setup.

This setup, shown in Figure 2, consisted of a board based on the Atmega328P micro-controller connected to a module based on the SX1278 integrated circuit (IC), which, among some other features, is a LoRa transceiver, and a RTL2832U-based USB software-defined radio (SDR) receiver (commonly referred to as RTL-SDR) connected to a PC. The first was programmed to transmit signals with the desired parameters and then they were received and recorded using the RTL-SDR and a simple flowgraph in GNU Radio to be subsequently decoded using a piece of software written on MATLAB/Octave developed for this study [18].

### 3.2. Previous Works

#### 3.2.1. General Transmitter Structure

**Figure 3:**
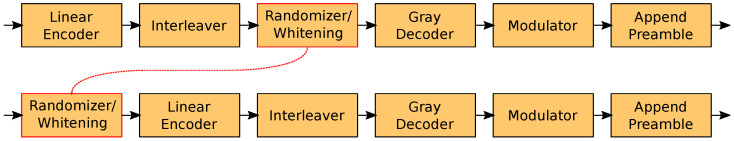
Transmitter structures, from previous works (**top**) and proposed (**bottom**).

In [2,3], similar structures for the LoRa receiver are proposed. Given those and the information provided in [16], we can summarize those structures as being composed of the following blocks connected sequentially, as shown in Figure 3.

After reverse engineering the whole protocol process, whose details will be described in the following sections, a new revised structure is proposed, shown in Figure 3.

- Linear Encoder
  The encoder encodes the bits with a (CR + 4,4) code, except for the data bits, which are always encoded with a (8,4) code.
  As mentioned above, the header bits are interleaved in a different manner than the payload bits. Actually, as mentioned in [3], it is the header bits plus the number of payload bits to complete 8 symbols, the 5 header code words plus SF-7 payload code words with 4-CR zeros added to the most significant bit to extend them to also have 8 bits, therefore resulting in 8*(SF-2) bits. They are then interleaved with a SF-2 interleaver, and the resulting symbol numbers are multiplied by 4, resulting in eight symbols in the $\left\{0;\;4;\;8;\;\ldots ;\;2^{SF}-4\right\}$ range. This enhances the robustness of the header bits against noise.
  The proposed linear encoder is the same as before, but in the following section on coding, a new, more intuitive description of the codes used is given.
- Interleaver
  This is a standard diagonal interleaver that turns a sequence of (CR + 4)-bit code words into a sequence of length SF-bit words, with the exception of the header bits that are interleaved with an 8-bit to (SF-2)-bit interleaver. The interleaver is kept as described in the literature.
- Randomizer/Data Whitening
  The randomizer takes as input two sequences of the same length: a pseudo-random binary sequence (referred to in [2,3] as a whitening sequence) and the output of the interleaver; the ouput is the result of the exclusive or operation (XOR) between the two sequences.
  This is the block which was changed the most; not only is its position changed in the receiver chain, but its structure is also fully reverse engineered and described more intuitively than before. Please note that the position of the randomizer was changed because in the previous works the whitening sequence needed to be reverse engineered for every set of parameters; therefore, an arbitrary sequence extracted from a commercial transmitter was applied. In our proposed solution, the randomizer adds (modulo-2 addition, i.e., exclusive or) a pseudo-random sequence of bytes generated by a linear-feedback shift register (LFSR) which is independent from the modulation parameters. Note that this sequence is only added to the payload bytes and not to the header or the payload “cyclic redundancy check (CRC)”.
- Gray Decoder
  The gray decoder turns the SF-bit (or (SF-2) if inside the header) words into symbol numbers. This structure is kept the same as described in the literature.
- Modulator
  The modulator generates the chirps corresponding to each symbol number. This structure is kept the same as described in the literature.
- Append Preamble
  This block adds a fixed preamble to the beginning of the resulting signal. This structure is kept the same as described in the literature, with the exception of a slight change on the size of the section of downchirp.

#### 3.2.2. Physical Layer Packet Structure

**Figure 4:**
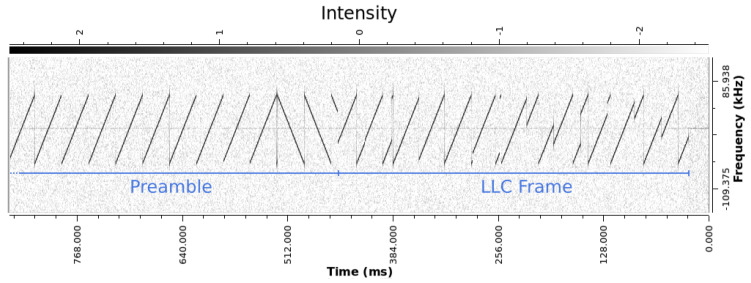
STFT of a section of a real LoRa signal.

The LoRa PHY packet is composed of a preamble, followed by LoRa PHY’s logical link control layer frame, modulated in CSS, as it can be seen in the plot of Short-time Fourier Transform (STFT) (Figure 4).

The preamble is composed of the following:

- A sequence of consecutive upchirps, which can be of length 6 to 65,535, as mentioned in [16];
- Two symbols, referred to as a sync word, used for network identification;
- Two and a quarter downchirps.

**Figure 5:**
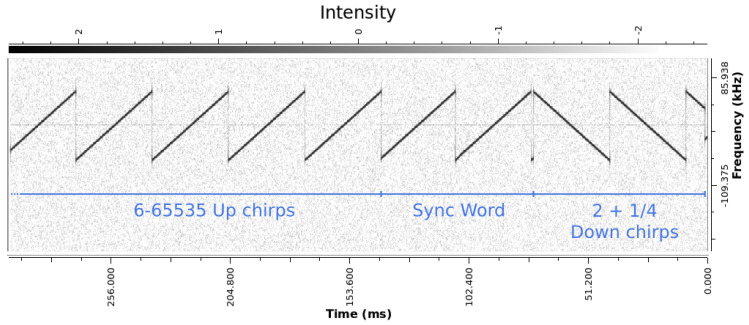
STFT of the preamble of a real LoRa signal.

A visualization of this signal can be seen in Figure 5, using a plot of the STFT of a real LoRa signal captured via SDR.

As previously mentioned in the section on previous works (Section 3.2), the packet is composed by the LLC payload preceded by a preamble. The structure described there seems to be correct, with a slight correction: the downchirps where found to be slightly shorter than two and a quarter. A length of 2 + 1/4 − 2^−SF^ symbols was determined empirically. The need for this change arose from observations during testing of the proposed receiver structure (see Section 4), as there was a systematic time offset in synchronization. Also, while using the receiver implemented in [19] that is based on the description from [3] for large frames (with payloads with length close or equal to 255 bytes) with a known input, and looking at how the received symbol numbers drift monotonically from the expected ones, we can see that there is some inaccuracy in the synchronization, further giving evidence to this hypothesis.

As a side note, this detail is probably the reason why the whitening/randomizer was not reverse engineered on previous papers.

#### 3.2.3. Logic Link Control Layer Structure

1. Coding
  Both [2,3] cite that the linear encoding used in LoRa PHY is some form of Hamming encoding and [3] explicitly describe it for CR = 4, as the usual form of Hamming(8, 4), mainly a code with generator matrix:
  $$\left(\begin{array}{cccccccc} 1 & 0 & 1 & 0 & 0 & 0 & 1 & 0\\ 0 & 1 & 1 & 0 & 0 & 1 & 1 & 0\\ 0 & 0 & 0 & 0 & 1 & 1 & 1 & 0\\ 1 & 1 & 1 & 1 & 1 & 1 & 1 & 1 \end{array}\right).$$
  With a permutation with the following permutation applied to the coded word:
  $$\left(\begin{array}{cccccccc} 5 & 0 & 1 & 2 & 4 & 3 & 6 & 7 \end{array}\right),$$
  represented in one-line notation. By applying the bit permutations to the generator matrices of the codes proposed in [2,3] and implemented in [19], in order to not need the extra bit-permutation step, new generator matrices were obtained. Also, the nibbles of the coded word were inverted to obtain a systematic code, which also resulted in a more intuitive structure of the randomizer, giving more evidence that this is indeed the intended bit ordering. For each of the possible CR values, mainly 1, 2, 3, and 4, the obtained generator matrices are listed in Equations (6)–(9), respectively, as follows:
  (6) $$\left(\begin{array}{ccccc} 1 & 0 & 0 & 0 & 1\\ 0 & 1 & 0 & 0 & 1\\ 0 & 0 & 1 & 0 & 1\\ 0 & 0 & 0 & 1 & 1 \end{array}\right);$$
  (7) $$\left(\begin{array}{cccccc} 1 & 0 & 0 & 0 & 1 & 0\\ 0 & 1 & 0 & 0 & 1 & 1\\ 0 & 0 & 1 & 0 & 1 & 1\\ 0 & 0 & 0 & 1 & 0 & 1 \end{array}\right);$$
  (8) $$\left(\begin{array}{ccccccc} 1 & 0 & 0 & 0 & 1 & 0 & 1\\ 0 & 1 & 0 & 0 & 1 & 1 & 1\\ 0 & 0 & 1 & 0 & 1 & 1 & 0\\ 0 & 0 & 0 & 1 & 0 & 1 & 1 \end{array}\right);$$
  (9) $$\left(\begin{array}{cccccccc} 1 & 0 & 0 & 0 & 1 & 0 & 1 & 1\\ 0 & 1 & 0 & 0 & 1 & 1 & 1 & 0\\ 0 & 0 & 1 & 0 & 1 & 1 & 0 & 1\\ 0 & 0 & 0 & 1 & 0 & 1 & 1 & 1 \end{array}\right).$$
  Note that all the obtained linear codes are systematic and with the exception of the first code, which is a simply a parity bit, the three others are all derived from Hamming(7,4). For CR = 3, the code is a systematic version of it, whereas for CR = 2 the code is a reduced version of this code obtained by removing the last bit, which results in a systematic (6,4) code; the last one (CR = 4) is a form of the extended Hamming(8,4) code obtained by adding an extra parity bit to the form of Hamming(7,4) used for CR = 3.
2. Randomizer
  Also referred to as a whitening block, the randomizer is partially reverse engineered in both [2,3] in a direct approach by assuming randomization is performed as the result of the XOR operation between a fixed arbitrary sequence and the output of the interleaver. These sequences were then extracted by sending all-zeros payload messages and looking at the resulting de-interleaved symbols, which should be the same as the whitening sequence. Note that, in this way, they depend on the modulation parameters (SF, CR, and the possible use of lowDataRate). These sequences can be found in  [19].
  The Berkelamp–Massey algorithm is an algorithm that finds the shortest LFSR that encodes a given sequence in GF(2), i.e., the Galois field of order 2. This approach is equivalent to solving the linear system that arises from the LFSR structure for a number of data points ($b_{i+N}=\sum_{i=0}^{N-1}b_{i-1-k}a_{k}$ for i∈{0,…,M−1}) using Gaussian elimination in GF(2). By taking the whitening sequences obtained in [3], used in [19], passing them through the decoder, grouping the obtained bits in a 8xN column bit matrix, and then running the Berkelamp–Massey algorithm on the first bytes, it can be seen that the sequence can be generated by a degree-8 LFSR with the following polynomial:
  (10) $$P\left(x\right)=x^{0}+x^{3}+x^{4}+x^{5}+x^{7}.$$
  The state of this LFSR is modulo-2 added to each of the data bytes to act as a randomizer. Furthermore, it is worth noting that the sequences provided by Robyns in [19] diverge from those generated by this LFSR after a certain position, but this is probably due to a symbol rate offset or an imprecision on the alignment that caused the last symbols of the message not to be aligned when using the receiver used in [19]. This conclusion was reached because the symbol numbers at the end of a 255-byte length frame slowly drift from the ones obtained in this work.
  It has to be pointed out that the whitening sequences proposed in [3] do work. Even with this problem, as the symbol number offsets caused by the time drift due to the inaccuracy in synchronization are deterministic and therefore can be compensated in the de-randomization process by changing the whitening sequence, like it was carried out unknowingly in [3].
3. Frame Structure
  In [3], the header structure seen in Table 3 is proposed as follows:

**Table 3:** General structure of the LoRa LLC.

Starting Bit	Function
0	Payload Length (1 byte)
8	CR (3 bits)
11	CRC Present (1 bit)
12	Header Checksum High Nibble (HN) (4 bits)
16	Header Checksum Low Nibble (LN) (4 bits)
20	Payload (0 to 255 bytes)
20 + 8 × (Payload Length)	Payload “CRC” (2 bytes) (optional)
  Each part of the frame is described in detail in the following sections.
  By analyzing the structure of the header, and given the previous description given in [3], the structure of the frame was found to be the one shown in Table 4.

**Table 4:** Proposed general structure of the LoRa LLC frame.

Starting Bit	Function
0	Payload Length HN (4 bits)
4	Payload Length LN (4 bits)
8	CR (3 bits)
11	CRC Present (1 bit)
12	Header Checksum HN (4 bits)
16	Header Checksum LN (4 bits)
20	Payload (0 to 255 bytes)
20 + 8 × (Payload Length)	Payload “CRC” (2 bytes) (optional) ^1^
20 + 8 × (Payload Length + 2)	Padding Nibbles ^2^

^1^ The payload “CRC” was found not to be in the usual ordering and not to be a usual CRC sum. Check its section for more details. ^2^ These padding nibbles are added so the encoding of the payload plus the payload nibbles plus the CRC (if present) plus these extra nibbles are a multiple of SF and therefore give rise, after the interleaver, to a whole number of symbols. How exactly these numbers are generated in real hardware is not clear, but they seem to make no difference as, while testing the implemented transmitter, they were set to zero, which is not what usually happens in the commercial transmitters; they still functioned normally.
  Note that the nibbles might seem to be inverted to that described in [3]. This is due to the change in the description of the coding.
  Each part of the frame is described in detail in the following sections:
  (a) Payload Length
    The length of the payload in bytes. Note that the two nibbles are in a “little endian-like” ordering with the upper nibble coming first and then the lower nibble.
  (b) CR
    The CR parameter of the LoRa PHY modulation as described in the Semtech documentation [20], which is not the code rate, but the difference between the code length and the code rank—this second always being equal to 4.
  (c) CRC Present
    Single bit that indicates whether or not the payload “CRC” is present. If it is ‘1’, then it is present, and if it is ‘0’, it is not.
  (d) Header Checksum
    A checksum calculated from the first 12 bits of the header. Its presence is mentioned in [3], but its exact structure is not mentioned—only that its 5 least significant bits are non-zero.
    The same “little endian-like” ordering used in the payload length was assumed. As described in Robyn’s work [3], 3 of the bits of the checksum are always 0, which in the assumed ordering are the last 3 bits (5 to 7). After doing some analysis, it was found that the checksum can be calculated as
    (11) c=Gh,
    where c is the bit column vector representation of the checksum, h is the vector of the first 12 bits of the header, and G is a 8×12 matrix in GF(2):
    (12) $$G=\left(\begin{array}{cccccccccccc} 1 & 0 & 0 & 0 & 0 & 1 & 0 & 0 & 1 & 1 & 1 & 1\\ 0 & 1 & 0 & 0 & 1 & 0 & 1 & 1 & 1 & 1 & 1 & 1\\ 0 & 0 & 1 & 0 & 1 & 0 & 1 & 0 & 0 & 1 & 0 & 1\\ 0 & 0 & 0 & 1 & 0 & 1 & 1 & 1 & 1 & 0 & 0 & 0\\ 1 & 1 & 1 & 1 & 0 & 0 & 0 & 0 & 0 & 0 & 0 & 0\\ 0 & 0 & 0 & 0 & 0 & 0 & 0 & 0 & 0 & 0 & 0 & 0\\ 0 & 0 & 0 & 0 & 0 & 0 & 0 & 0 & 0 & 0 & 0 & 0\\ 0 & 0 & 0 & 0 & 0 & 0 & 0 & 0 & 0 & 0 & 0 & 0 \end{array}\right).$$
    - Reverse Engineering Methodology
      In order to reverse engineer this checksum, initially, the reasonable assumption that the bits of the header checksum is a linear function (in modulo-2 arithmetic) of the data bits of the header was made. Then, by decoding the headers of frames from captured waveform files, putting them as rows of a matrix, and then applying the Gauss–Jordan elimination algorithm in modulo-2 arithmetic. In this way, if we start with a matrix with 12 linearly independent rows (in the canonical linear space in GF(2)^12^), we obtain a matrix M, such that
      (13) $$M=\left[I\;G^{⊺}\right],$$
      i.e., M is the result of the horizontal concatenation between the transpose of G and the 12×12 identity matrix I.
  (e) Payload “CRC”
    The CRC sum of the payload. It is mentioned in [2,3], but its exact structure was not known.
    The payload “CRC” was found to be calculated by taking the polynomial in GF(2) relative to the payload data in little endian ordering, taking the remainder of its division with the polynomial $x^{0}+x^{5}+x^{12}+x^{16}$, and then taking the corresponding bit string and storing it in big endian ordering. Note that this division is equivalent to computing the CRC sum of the data starting from the second byte (in little endian order) with the above-mentioned polynomial and using the first byte as the initial value of the algorithm.
    - Reverse Engineering Methodology
      Initially, given what was already known, it was assumed that the CRC sum was really a CRC sum and had a degree-16 polynomial. In order to reverse engineer the payload CRC, using the above-mentioned test setup, frames whose payload was the powers of two, i.e., only one ‘1’ byte and all the others ‘0’, with length 1 up to 7 were transmitted, captured, and decoded. For the sake of simplicity, all byte orderings referred to in the rest of this description are big endian. When observing the data, it can be seen that when only the last 2 bytes are non-zero, the CRC is the same as those last 2 bytes, but in opposite ordering. Given this, three things were inferred as follows:
      - The byte ordering of the CRC was the opposite of that of the data.
      - The checksum was not a direct CRC sum, but the direct remainder of the polynomial division of the data without multiplying its polynomial by x^n^, where n is the order of the CRC.
      - The data used in this calculation is in little endian order because the CRC sum was the same as the data but inverted when only two non-zero bytes are transmitted.
      Given those three, if the last bytes of the payload are zero, the checksum is equivalent to a usual CRC sum of the data in little endian mode, ignoring those last two bits. Therefore, it was possible to use the open-source tool CRC RevEng [21] to find the polynomial if this checksum was indeed a CRC. This program tests a collection of known used CRC polynomials and checks if any are consistent with the given data plus CRCs. By running it with payload data, the program yielded the polynomial 0x1021 and indicated that the data was indeed taken in little endian ordering. For further testing, with all power-of-two length payloads, it was verified that this CRC coincided with the one calculated by the commercial transceiver for payload lengths 1 up to 7 bytes.

## 4. Receiver Structure

As the transmitter structure was already described in Section 3.2.1 to illustrate the structure of the frame, in this section, a structure for the receiver is proposed. The receiver architecture can be divided into four main blocks:

- Frequency Estimator
- Correlation Synchronizer
- Symbol Decision
- Frame Decoder

### 4.1. Frequency Estimator

The goal of this block is to estimate the instantaneous frequency of the signal, given some known chirp rate, or equivalently, a symbol factor and a CSS bandwidth. This can be formally defined as finding, for each time instant n, some $f\in ]-\frac{1}{2};\frac{1}{2}]$, such that minimizes

(14) $$J\left(f,n\right)=E\left[{\left|\sum_{i=-N}^{N}x\left(i+n\right)\cos\left(\pi \beta i^{2}\right)e^{j2\pi fi}\right|}^{2}\right],$$

or, equivalently, that maximizes

(15) $$K\left(f,n\right)=E\left[{\left|Y_{n}\left(f\right)\right|}^{2}\right]$$

with

(16) $$Y_{n}\left(f\right)=\sum_{i=-N}^{N}x\left(i+n\right)\cos\left(\pi \beta i^{2}\right)e^{-j2\pi fi}=\mathrm{DTFT}\left[{\left(x\left(i+n\right)\cos\left(\pi \beta i^{2}\right)\right)}_{i\in \{-N,\ldots ,N\}}\right],$$

where, for some N∈N, where x(i) is the received signal that consists of the LoRa signal added to a zero-mean white noise process n(i) with variance $\sigma_{n}^{2}$, while DTFT denotes the discrete-time Fourier transform.

Note that $\cos\left(\pi \beta i^{2}\right)=\frac{1}{2}\left(e^{j\pi \beta i^{2}}+e^{-j\pi \beta i^{2}}\right)$ is used instead of $e^{-j\pi \beta i^{2}}$, as the LoRa CSS signal uses not only upchirps, but also downchirps, the second for synchronization, as described in the section about the modulation; by using the sum of a downchirp and an upchirp, when multiplied by this reference signal, the signal vector will present a peak on its spectrum at its middle frequency when either chirp is present.

To estimate this value, two approaches are proposed.

**Figure 6:**
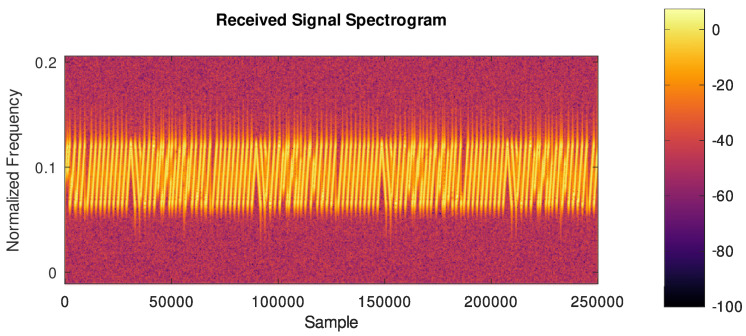
Spectrogram of the input signal composed of a sequence of LoRa frames with SF = 7, CRC on, and payloads of a single byte containing powers of 2 (1, 2, …, 128).

1. Stochastic Gradient Descent
  If it is assumed there is no interfering signal other than Gaussian noise, this problem can be approximated with a simpler optimization problem using only one sample, i.e., to minimize the cost function:
  (17) $$J\left(w\right)=E\left[{\left|x\left(n+1\right)e^{-j2\pi \beta }-w^{*}x\left(n\right)\right|}^{2}\right],w\in C,$$
  using $\hat{f}=-\frac{arg(w)}{2\pi }$ as the instantaneous angular frequency estimate. The solution of this problem can be easily estimated using stochastic gradient descent, i.e., an order-1 adaptive filter. For the reverse engineering carried out in this work, an order-1 normalized least mean squares filter was used whose coefficient-update function reduces to
  (18) $$w\left(n+1\right)=w\left(n\right)\left(1-\mu \right)+\mu {\left(\frac{x\left(n+1\right)e^{-j2\pi \beta }}{x(n)}\right)}^{*},\mu \in R^{+},$$
  which is equivalent to passing the signal $\frac{e^{-j2\pi \beta }x_{i+1}}{x_{i}}$ through a single pole IIR low pass filter.
  Also, to avoid division by zero errors, the alternative equation
  (19) $$w\left(n+1\right)=w\left(n\right)\left(1-\mu \right)+\mu \;\mathrm{sgn}\left(e^{-j2\pi \beta }x{\left(n+1\right)}^{*}x(n)\right),\mu \in R^{+}$$
  can be used.
  An example of the output signal of this block, in which the input signal is composed of a sequence of LoRa frames with SF = 7 whose spectrogram is shown in Figure 6, can be seen in Figure 7.

**Figure 7:**
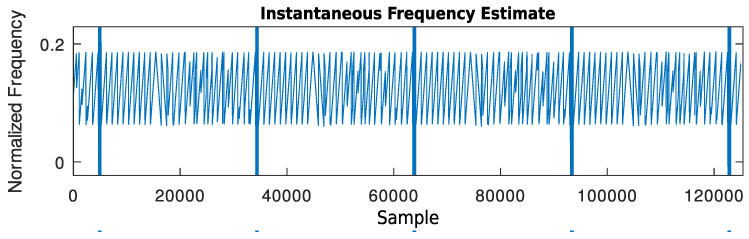
Output signal of the stochastic gradient descent frequency tracker given the described test signal at its input (zoomed into frequencies from 0 to 0.2).
2. Discrete Fourier Transform (DFT) Peak
  The previous method presents some problems as if multiple local maxima are present in the short-time spectrum, e.g., if there is an interfering signal, the frequency estimate will converge to an intermediate solution that minimizes the square error of the simplified cost function and not the global maximum that optimizes the original function.
  If the squared magnitude of the DTFT of the signal windowed by a (2N+1)-sample rectangular window around each sample is seen as the probability density of the instantaneous frequency process, the original problem can be interpreted as finding its mode, whereas the simplified process converges to its mean; therefore, it is only working if the interfering signal’s mean is equal to its mode, as is the case for a signal with Gaussian interference.
  To cope with this problem, we can use the following estimator for the frequency:
  (20) $$\hat{f}\left(n\right)=\frac{1}{N}\underset{k}{arg max}\left({\left|Y_{n}\left(k\right)\right|}^{2}\right),$$
  where
  (21) $$Y_{n}\left(k\right)=\mathrm{DFT}{\left[{\left(x\left(i+n\right)\cos\left(\pi \beta {\left(i-\frac{N-1}{2}\right)}^{2}\right)\right)}_{i\in \{0,\ldots ,N-1\}}\right]}_{k},$$
  which is equivalent to computing the original cost function at N points and taking the point that maximizes it.
  Additionally, to compute the cost function at more frequency points, the order of the DFT can be increased while applying a small rectangular window to the signal, i.e.,
  (22) $$\hat{f}\left(n\right)=\frac{1}{N^{\prime }}\underset{k}{arg max}\left({\left|Y^{\prime }\left(k\right)\right|}^{2}\right)\;,$$
  with
  (23) $$Y^{\prime }\left(k\right)=\mathrm{DFT}{\left[{\left(x\left(i+n\right)w\left(i\right)\cos\left(\pi \beta {\left(i-\frac{N^{\prime }-1}{2}\right)}^{2}\right)\right)}_{i\in \{0,\ldots ,N^{\prime }-1\}}\right]}_{k}\;,$$
  where
  (24) $$w\left(i\right)=\left\{\begin{array}{cc} 1 & \mathrm{if}\;\left|i\right|\leq N^{\prime }\\ 0 & \mathrm{if}\;|i|>N_{w} \end{array}\right.$$
  and $N_{w}\leq N^{\prime }$.
  This method presents some issues in terms of computational cost; however, it has much better performance in terms of resistance to interference from signals with different symbol factors [10], as multiplying the signal by $cos(\pi \beta i^{2})$ spreads the spectrum of the interfering signals and collapses the desired signal to a single (or two, in the case of the CSS modulation at the transition of two symbols) peak.
  To address the performance issues, the $\hat{f}\left(k\right)$ estimator can be computed at a lower rate than the input signal, i.e.,
  (25) $$\hat{f}\left(n\right)=\frac{1}{N^{\prime }}\underset{k}{arg max}\left({\left|Y^{\prime \prime }\left(k,d\right)\right|}^{2}\right),d\in N\;,$$
  where
  (26) $$Y^{\prime \prime }\left(k,d\right)=\mathrm{DFT}{\left[{\left(x\left(i+dn\right)\cos\left(\pi \beta {\left(i-\frac{N^{\prime }-1}{2}\right)}^{2}\right)\right)}_{i\in \{0,\ldots ,N^{\prime }-1\}}\right]}_{l}\;.$$
  Just as in the previous method, an example of output of this block when receiving the beginning of the same test signal is shown in Figure 8.

### 4.2. Correlation Synchronizer

This part is split into three sections: the calculation of the correlation, the actual synchronization/alignment, and the compensation.

#### 4.2.1. Correlator

The correlation is calculated by taking the most recent $N_{pr}$ samples of the instantaneous frequency generated by the frequency estimator, where $N_{pr}$ is the size of the fixed part of the preamble, i.e., the sync word plus the downchirps and computing its normalized correlation with the expected preamble, i.e.,

(27) $$c\left(i\right)=\frac{{\hat{f}}_{n}\cdot p_{n}}{∥{\hat{f}}_{n}∥∥p_{n}∥},$$

where ${\hat{f}}_{n}={\left[\hat{f}\left(n\right),\cdots ,\hat{f}\left(n-N_{pr}+1\right)\right]}^{H}$ and ${\left[\cdot \right]}^{H}$ denotes the Hermitian transpose.

Then this value and the first sample of the input vector are passed on to the next stage.

**Figure 9:**
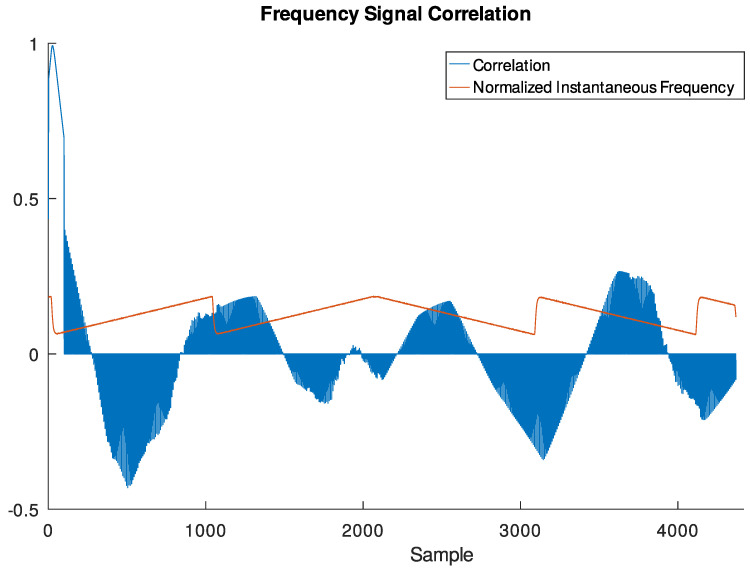
Output signals of the correlator when receiving the end of the preamble of a LoRa frame with SF = 7, CRC on, and payload of a single byte containing a power of 2 (1, 2, …, 128).

An example of the outputs of this block when receiving the end of the preamble of a LoRa PHY frame is given in Figure 9:

#### 4.2.2. Synchronizer

This block takes the two values generated by the correlator and looks for a local maximum, given two thresholds. When the correlation is higher than the first threshold, it starts trying to find the local maximum, and when the correlation gets lower than the second threshold, it stops this detection and outputs the signal starting from the point of maximum correlation between these two instants, already grouped in vectors of size $n_{sym}$, i.e., the number of samples in a symbol.

**Figure 10:**
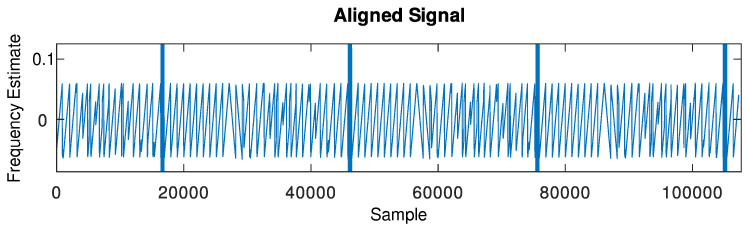
Synchronized signal, as generated by the synchronizer when its input is the previously shown test signal.

The resulting output, when the previously shown output of the correlator is the input of the synchronizer, can be seen in Figure 10.

#### 4.2.3. Time-Frequency Shift Compensation

In this step, two parameters are estimated and compensated by adding an offset to the instantaneous frequency signal: the frequency offset, i.e, the mean frequency of the signal and the fractional time offset, i.e, the remaining non-integer time offset of the signal after synchronization.

Initially, the two sync word symbols and the two downchirps are demodulated using a method similar to that described in the section on the multiple detection approach (Section 4.3.2) for symbol detection, with the difference that the sync word samples are subtracted by the expected sync word and the downchirps are demodulated considering downchirp symbols. Also, two obtained new sync word symbols are demodulated together generating a single offset value and the same is carried out for the two downchirps.

**Figure 11:**
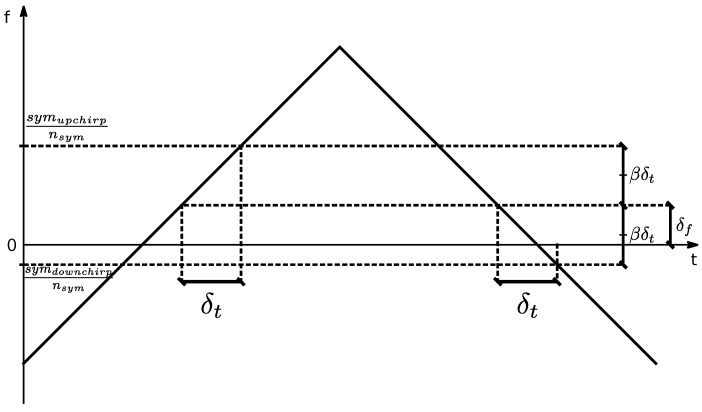
Illustration of the offset estimation procedure with a single upchirp (as sync word) and downchirp.

With these two values in hand, their average scaled by $\frac{1}{n_{sym}}$ gives an estimate of the frequency offset, and their difference scaled by $\frac{1}{2\beta n_{sym}}$ gives an estimate of the fractional time shift, as illustrated in Figure 11:

(28) $$\delta_{f}=\frac{sym_{upchirps}+sym_{downchirps}}{2n_{sym}},$$

(29) $$\delta_{t}=\frac{sym_{upchirps}-sym_{downchirps}}{2\beta n_{sym}}.$$

Finally, the calculated frequency offset is subtracted from the frequency estimates signal, and, to avoid complex fractional resampling operations, the time offset is compensated by adding an extra equivalent offset:

(30) $$\delta_{f,t,eq}=\beta \delta_{t},$$

(31) $$\delta_{f,tot}=\delta_{f}+\delta_{f,t,eq},$$

(32) $$f_{comp}\left(n\right)=f\left(n\right)-\delta_{f,total},$$

where β is the chirp rate relative to the current modulation parameters. This relies on the local linearity of the frequency-time waveform, which allows the time offset to be compensated by shifting in frequency.

### 4.3. Symbol Decision

In a similar manner to the frequency estimator, two methods of symbol demodulation were proposed as follows: one based on the mean instantaneous frequency of the symbol being detected, and one based on the mode.

#### 4.3.1. Minimum Squares Approach

This method, based on the one described in [3], incorporates the previously mentioned frequency estimation techniques. It determines the symbol by calculating the inner product between the symbol’s frequency samples and each expected symbol, selecting the one that maximizes this result. Since the symbols are rotations of one another, this process can efficiently be achieved by computing the circular correlation with the 0-th symbol, i.e., the time-frequency representation of the base upchirp. This approach significantly reduces the computational complexity from $O\left(n_{sym}^{2}\right)$ to $O\left(n_{sym}\log_{2}\left(n_{sym}\right)\right)$, where $n_{sym}$ represents the number of frequency samples per symbol in a DFT-based approach.

The main advantage of this approach is that it is insensitive to both frequency offsets and frequency scaling, as the instantaneous frequency vector of the base upchirp has zero mean.

**Figure 12:**
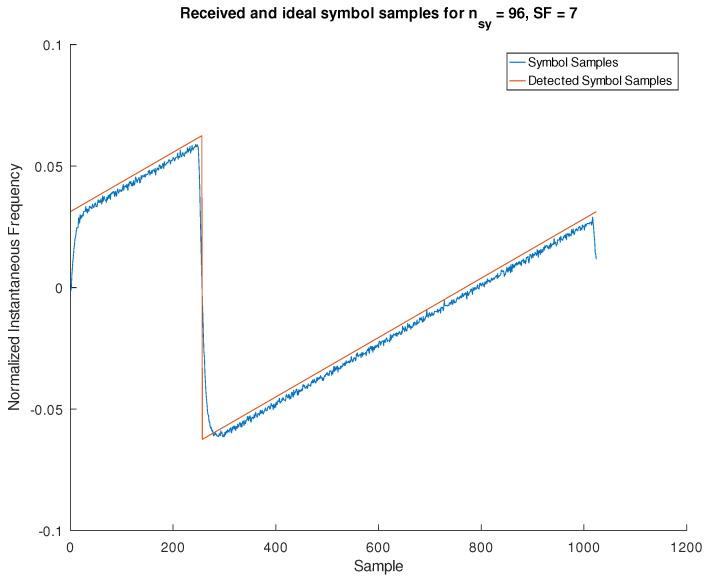
Synchronized frequency samples of a received LoRa symbol together with the samples of the symbol detected by the minimum squares decider block when given the former as an input.

In Figure 12, we show an example of the vector used as input to the symbol decision block together with the samples of the decided symbol.

#### 4.3.2. Multiple Detection Approach

The previous method assumes the interfering signal has zero mean frequency within the observation window, which is not necessarily true when using the DFT-based approach for frequency estimation, as the behavior of the estimate in the transition is not very predictable. In this region, there are two spectral peaks with magnitudes close two each other; therefore, the maximum will alternate between them. To cope with this problem, a new method is proposed that makes a decision for each of the points of the symbol and takes the most frequent one using the following estimator:

(33) $$s_{i}=\mathrm{mod}\left(\left[\left({\hat{f}}_{i}-\beta \left(i-\frac{n_{sym}-1}{2}\right)\right)\frac{2^{SF}}{BW}\right],2^{SF}\right),$$

where the brackets represent the nearest integer rounding and ${\hat{f}}_{i}$ is the vector of frequencies estimated for the current symbol.

The main issue with this method is that it requires time-frequency alignment like the procedure described in Section 4.2.3 to be performed beforehand.

Also, to improve performance, only the middle half of the samples of the frequency waveform are used, i.e., samples at instants $i\in \{\frac{n_{sym}}{4},\ldots ,\frac{3n_{sym}}{4}-1\}$. This also helps avoid interference from neighboring symbols at the edges of the symbol due to time shifts.

### 4.4. Frame Decoder/Receiver Controller

This section implements the inverse of the steps described in Section 3.2.1, reads the frame header, and performs the CRC check if needed. It executes the following operations in order as follows:

- Gray Encoding
- Deinterleaving
- Decoding
- Randomization
- CRC Calculation

## 5. Transceiver and Hardware Implementation

### 5.1. GNU Radio Implementation

For the implementation of the receiver, GNU Radio was used. It is a free and open-source software development toolkit used for the development of signal processing blocks that can be connected in a flowgraph. Among all signal processing software development toolkits, the choice of GNU Radio was based on its flexibility, customizability, and compatibility with very well-known SDR platforms. Please note that all the processing blocks work in baseband in the host computer environment, as this is the main idea of SDR processing. These custom blocks can be written in C++ or Python and the flowgraphs can be either created using the GNU Radio Companion graphical interface and then exported to the above-mentioned languages or directly written in those. All the GNU Radio-based code developed for this study can be found at  [22,23]. Please note that both the SDR-based receiver and transmitter were tested and validated using a commercial LoRa IC, the SX1278, to guarantee that they can communicate with original LoRa hardware.

#### 5.1.1. Receiver

In order to keep the design modular, not only so it can be more easily modified, but also to take advantage of the multi-threading capabilities GNU Radio provides, as each block runs in a separate thread, each of the sections described in the previous section have been implemented in separate blocks.

In addition to these, two extra blocks were added as follows:

- A receiver controller that, in order to control the flow of data and pass some necessary information between the blocks, controls some aspects of all other blocks using message ports, which are an asynchronous way of passing information between blocks that GNU Radio provides.
- A chirp detector that, using an approach similar to that described in the DFT-based symbol decision, takes the DFT of the signal multiplied by the linear chirp relative to the selected set of modulation parameters (BW_CSS_, SF, and consequently the chirp rate), computes the chirp-windowed DFT and the ratio between the energy in the maximum bin and the mean energy on the remaining bins, and checks whether this value is higher than some set threshold. Then, it uses this information to only allow the flow of data downstream in the flowgraph when this detection happens in order to avoid unnecessary calculations and consequently unnecessary power consumption.

**Figure 13:**
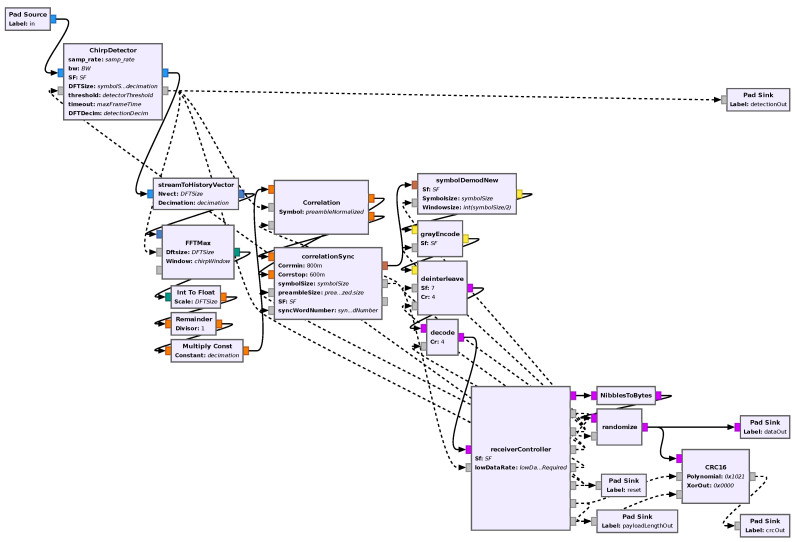
Flowgraph of the receiver in GNU Radio companion.

Finally, all these blocks were connected together in a hierarchical flowgraph to create a receiver block. An image of the flowgraph of the receiver is shown in Figure 13.

#### 5.1.2. Transmitter

The transmitter follows the same design approach as the receiver; that is, it follows the proposed structure and tries to be as modular as possible. Also, in addition to what was previously described, two extra blocks were added as follows:

- A transmitter controller that controls all other blocks by setting the required parameters, according to the parameters given to the transmitter, and depending on which part of the packet is being transmitted. This block, differently to the controller in the receiver, mainly uses tags to control the blocks to keep the implementation cleaner (tags are another mechanism of asynchronous message passing on GNU Radio that embeds itself into existing data streams instead of requiring an extra output in the block). Also, this block (together with the other blocks) supports dynamically setting all modulation parameters (SF, CR, BW, payload size) by sending a special tag to it.
- The append silence block. This is needed because of how GNU Radio works and how most stock sink blocks are implemented, a continuous stream of data is required at the output of the receiver; this block is controlled by the transmitter controller and generates silence samples (i.e., of value zero) whenever the rest of the blocks are not outputting any packets and outputs that data when it is available.

**Figure 14:**
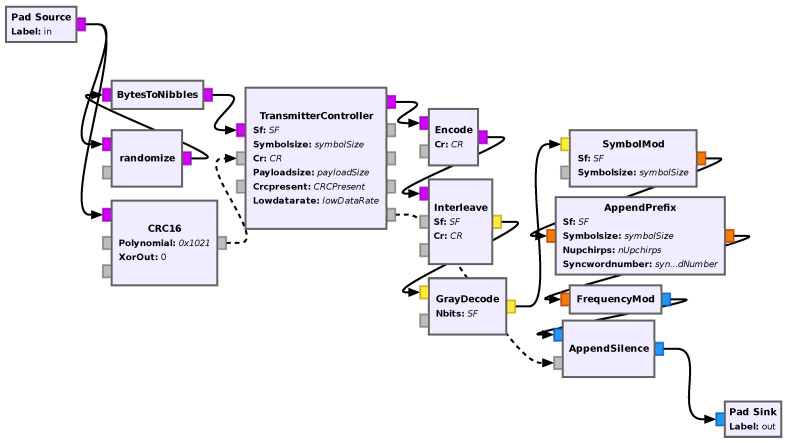
Flowgraph of the transmitter in GNU Radio companion.

An image of the flowgraph of the receiver is shown in Figure 14.

In addition to the blocks themselves, another change was introduced. As it is not trivial in GNU Radio to instantiate multiple instances of the receiver and use them with a single sink, a mechanism to update the parameters of the transmitter via tags sent through its input was implemented, so a single block can be used and receive data with different modulation parameters to be controlled by external blocks.

### 5.2. SDR Platforms and Hardware Setup

Since its introduction by Mitola [24], the software-defined radio concept has revolutionized the approach of testing and prototyping in the wireless communication field. The SDR concept is based on the fact that components, which conventionally were implemented in analog hardware, are now instead implemented by means of software on a computer or embedded system. This approach allows the re-use of the same hardware for different purposes and communication systems; it is sufficient to change the software on the computer. Table 5 shows a short list of the most well-known SDR platforms [25,26] available in the market. The Universal Software Radio Peripheral (USRP) was one of the first SDR platforms to gain popularity thanks to its seamless integration with GNU Radio. Current USRP models B210 and X310 are mid- to high-level SDR transceivers that can offer very high sample rates, large bandwidths, and high speed interfaces with a cost of a few thousand dollars.

**Figure 15:**
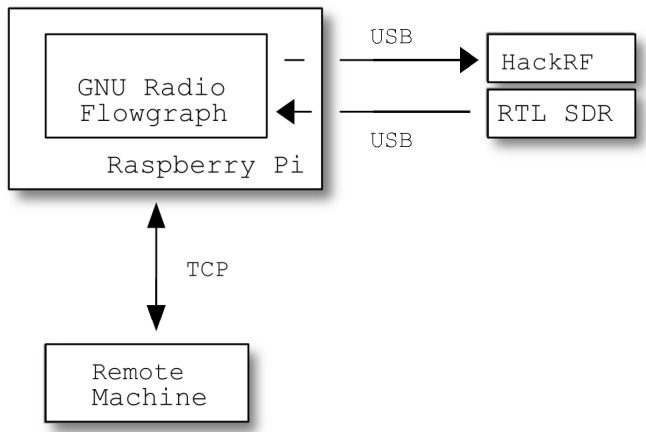
Block diagram of employed hardware setup.

For IoT narrowband applications, the required bandwidth and sample rate are much less stringent; therefore, for our experiment, we used an RTL-SDR (RTL2832U chipset) to work as a receiver and a HackRF One SDR transceiver to work as a transmitter. The main drivers behind this choice were the relative low cost and wide availability of these devices. The hardware setup was then completed with a Raspberry Pi 3A+ as the computer host to run GNU Radio on it. A simple diagram depicting this setup is shown in Figure 15.

## 6. Example Applications

We will now showcase how the developed library can be used, not only to implement the functionality of a simple transceiver, but also how it can be employed in more complex applications that take advantage of the flexibility built into the blocks.

### 6.1. LoRa Detector: Another Application of the Chirp Detector

**Figure 16:**
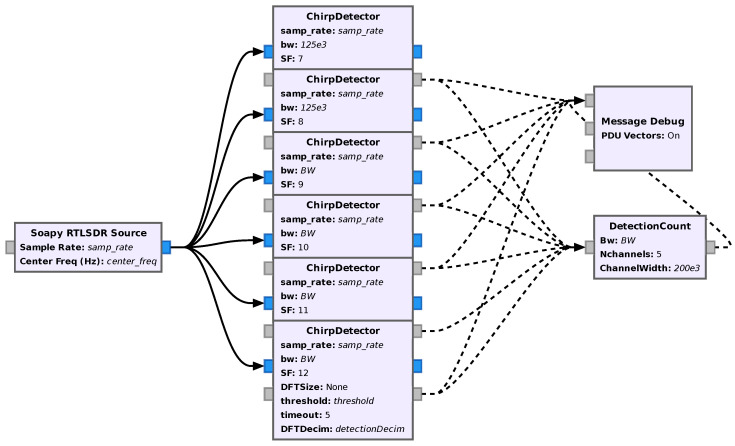
Flowgraph of the LoRa multi-channel multi-SF detector.

With some simple modifications, the above-mentioned Chirp Detector (in Section 5.1.1) can also be used for detecting the presence of different SF signals in multiple channels. This is especially useful, as it allows us to obtain useful statistics about the local channel/network without all the computational resources required by multiple receivers running in parallel. This was carried out by making its data output optional and adding a message output port that, whenever it detects a transmission, is used to send out a message containing the parameters of the block (SF, BW, and sample rate) and the normalized center frequency of the band where the detection happened. In this way, by running multiple of these detectors in parallel while keeping the sample rate high enough so multiple channels can be observed simultaneously and sending the messages they generate to a block that receives and interprets these messages, one can easily perform statistics on channel usage for each SF and band. In order to demonstrate this, a simple block that takes these messages and counts the detected transmissions on each channel-SF pair was developed. An image of the entire flowgraph developed for this application is shown in Figure 16.

Also, it is worth pointing out that this extra information that is output by the chirp detectors could be used in future implementations for controlling the center frequency of a filter to which the input signal is fed, making it possible to select the channel in which data is being transmitted with a certain SF and BW and forward it to an appropriate receiver, thus allowing for the implementation of an efficient multi-channel receiver system.

### 6.2. Multi-Parameter, Multi-Channel Receiver

**Figure 17:**
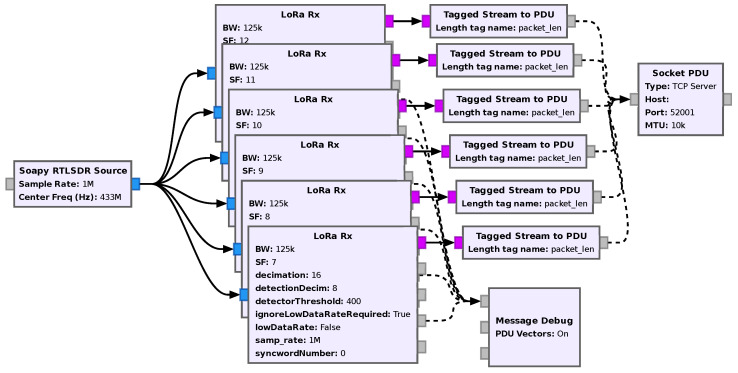
Flowgraph of the multi-parameter, multi-channel receiver.

In this example application, five receivers are run in parallel, each with a different spreading factor and with a sample rate of 1 MS/s, as shown in the flowgraph of Figure 17 In this way, any signal transmitted in any channel within a 1 MHz band with any spreading factor can be received.

In addition, in order to allow simple integration with other applications, a TCP interface is also added. In this particular setup, one can run the receiver at a dedicated Raspberry Pi 3A+ and receive the data via TCP on any network-connected device.

### 6.3. Variable Parameter Transmitter

In order to make the transmitter block able to have its parameters changed during runtime, an interface based on GNU Radio’s tag propagation mechanism was implemented. This allows for metadata containing modulation and band parameters to be optionally propagated together with the data stream in order to change the parameters when needed.

**Figure 18:**
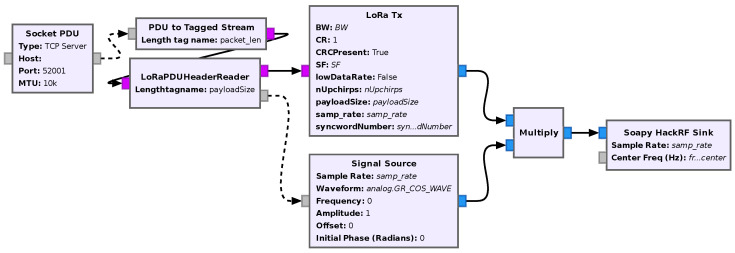
Flowgraph of the variable parameter transmitter.

In addition, an interface to translate a packet with a special format to GNU Radio tags, extract the band information, if present, and generate a message to control a GNU Radio signal source in order to select the band was also added to allow the transmitter to be controlled by external devices via network. The packet consists of this structure:

fullflexible
struct loraPDUHeader {
int8_t hasHeader;
int8_t SF;
uint8_t CR;
bool payloadCRCPresent;
bool lowDataRate;
float BW;
uint8_t syncWordNum;
float fOffset;
};

followed by the actual payload data to be transmitted via the LoRa transmitter. The hasHeader field is always 0x01 if a header with configuration is present; therefore, if one is not present, the user should send a single byte before the payload data with any value other than 0x01. The flowgraph of the variable parameter transmitter is shown in Figure 18.

### 6.4. Multi-Parameter, Multi-Channel Transceiver

**Figure 19:**
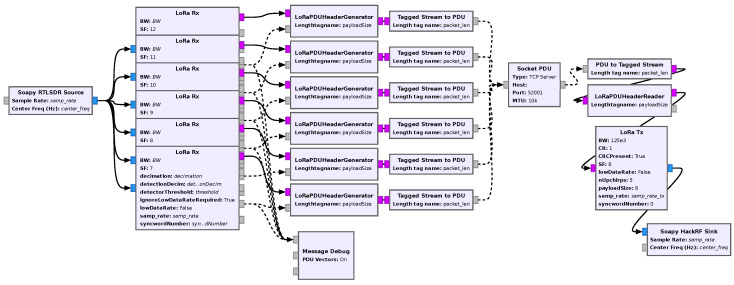
Flowgraph of an example of the full multi-parameter, multi-channel transceiver.

By joining the two previously mentioned flowgraphs, a full transceiver, which can both receive and transmit with multiple modulation parameters and channels controlled by a TCP interface, was implemented, and its flowgraph is shown in Figure 19. The idea behind this is that it could potentially be used for implementing fully functional LoRaWAN nodes and even gateways with the physical and logical link control layers, i.e., LoRa PHY, running in a remote device, and the MAC layer protocols running in the local sender device. However, it is worth noting that, in its current state, this application cannot be run stably in the Raspberry Pi 3A+ due to random-access memory (RAM) speed and size limitations, but it is very likely that with some extra optimization effort, it could be made to run successfully in this device.

## 7. Conclusions

In this paper, we proposed our contribution on reverse engineering the lower layers of LoRa, commonly referred to as LoRa PHY, we proposed a structure for its transceiver and implemented this structure by using the GNU Radio software to be in conjuction with widely available software-defined radio platforms. With regard to the reverse engineering of LoRa PHY, all the missing details were revealed, together with the methodology used to find them, and we thoroughly clarified how this part of this protocol stack works. For the transceiver structure, new demodulation and synchronization methods were proposed, which potentially bring better interference resistance performance with respect to the previously proposed methods. As far as the actual implementation of the transceiver is concerned, not only the feasibility of the proposed methods were tested with real hardware, but also a completely free and open-source LoRa PHY transceiver implementation was made available to serve as foundation for further research and development on this protocol stack. Also, an example of hardware implementation was proposed and tested, showing how this implementation of the transceiver can be used, extended, and integrated by exploiting the capabilities of the GNU Radio library. As a final remark, some technical details and challenges still remain to be solved in future research, mainly to optimize the transceiver algorithms and code for it to be able to run in even simpler hardware, to increase its range of applications and to implement features which were not included in this study, such as the support for LoRa’s implicit mode.

## Figures and Tables

**Figure 8:**
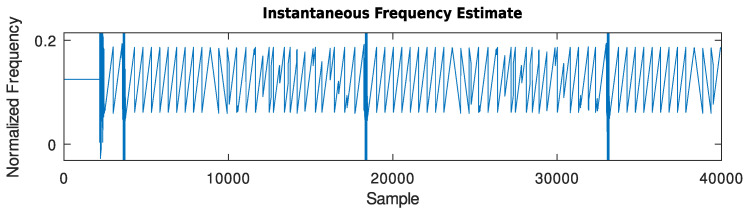
Output signal of the DFT peak frequency tracker given the described test signal at its input.

**Table 1:** Comparison of selected characteristics of LoRa, NB-IoT, and Sigfox.

LoRaWAN + LoRa PHY	
Band	Industrial, Scientific, and Medical (ISM)
Modulation	Chirp Spread Spectrum (CSS) (Section 2.3)
Bandwidth	125, 250, or 500 kHz [9]
Physical Bit Rate	0.37–27.4 kbit/s (See Section 2.3)
Multiplexing	Different-SF Chirp Interference Resistance (See [10])
Channel Access	Deterministic Time Slots [1]
**NB-IoT**	
Band	Same as LTE
Modulation	BPSK/QPSK [11]
Bandwidth	3.75 or 15 kHz [11]
Physical Bit Rate	3.75, 7.5, 15 or 30 kbit/s [11]
Multiplexing	Single Carrier Frequency Division
	Multiple Access with Frequency Hopping [12]
Channel Access	Contention-based random access procedure [12]
**Sigfox**	
Band	ISM
Modulation	Differential BPSK [13]
Bandwidth	0.1 kHz or 0.6 kHz [13]
Physical Bit Rate	0.1 kbit/s or 0.6 kbit/s [13]
Multiplexing	Random Frequency Time-Division Multiple Access [13]
Channel Access	Deterministic Time Slots [14]

**Table 2:** Physical bit rate values (in kbit/s) for selected combinations of BW and SF ($R_{b}=BW\cdot 2^{-SF}\cdot SF$).

	SF	7	8	9	10	11	12
BW (kHz)							
125		6.84	3.91	2.2	1.22	0.67	0.37
250		13.67	7.81	4.39	2.44	1.34	0.73
500		27.34	15.63	8.79	4.88	2.69	1.46

**Table 5:** List of SDR platforms.

Platform	Frequency Range	Maximum Sampling Rate	TX/RX?	Interface	Price
RTL-SDR RTL2832U	24 MHz–1.7 GHz	2.56 Msps	Only RX	USB	USD 10
DVB-T TV Tuner					
HackRF One	1 MHz–6 GHz	20 Msps	Yes	USB 2.0	USD 300
LimeSDR	10 MHz–3.5 GHz	30.72 Msps	Yes	USB 3.0	USD 400
Mini 2				PCIe	
USRP B210	70 MHz–6 GHz	56 Msps	Yes	USB 3.0	USD 1100
USRP X310	DC-6 GHz	200 Msps	Yes	GbE	USD 4800
				PCIe	

## Data Availability

The original data presented in the study are openly available in GitLab at https://gitlab.com/jpsimas/librelora.
